# A hierarchical anatomical classification schema for prediction of phenotypic side effects

**DOI:** 10.1371/journal.pone.0193959

**Published:** 2018-03-01

**Authors:** Somin Wadhwa, Aishwarya Gupta, Shubham Dokania, Rakesh Kanji, Ganesh Bagler

**Affiliations:** 1 Center for Computational Biology, Indraprastha Institute of Information Technology (IIIT-Delhi), New Delhi, 110020, India; 2 Maharaja Agrasen Institute of Technology, Guru Gobind Singh Indraprastha University, New Delhi, 110086, India; 3 Indira Gandhi Delhi Technical University for Women, New Delhi, 110006, India; 4 Delhi Technological University, New Delhi, 110042, India; 5 Department of Bioscience and Bioengineering, Indian Institute of Technology Jodhpur, Jodhpur, 342011, India; Chuo University, JAPAN

## Abstract

Prediction of adverse drug reactions is an important problem in drug discovery endeavors which can be addressed with data-driven strategies. SIDER is one of the most reliable and frequently used datasets for identification of key features as well as building machine learning models for side effects prediction. The inherently unbalanced nature of this data presents with a difficult multi-label multi-class problem towards prediction of drug side effects. We highlight the intrinsic issue with SIDER data and methodological flaws in relying on performance measures such as AUC while attempting to predict side effects.We argue for the use of metrics that are robust to class imbalance for evaluation of classifiers. Importantly, we present a ‘hierarchical anatomical classification schema’ which aggregates side effects into organs, sub-systems, and systems. With the help of a weighted performance measure, using 5-fold cross-validation we show that this strategy facilitates biologically meaningful side effects prediction at different levels of anatomical hierarchy. By implementing various machine learning classifiers we show that Random Forest model yields best classification accuracy at each level of coarse-graining. The manually curated, hierarchical schema for side effects can also serve as the basis of future studies towards prediction of adverse reactions and identification of key features linked to specific organ systems. Our study provides a strategy for hierarchical classification of side effects rooted in the anatomy and can pave the way for calibrated expert systems for multi-level prediction of side effects.

## Introduction

Drugs produce a myriad of effects by virtue of their interactions with cellular mechanisms. Beyond their therapeutic effects, drugs are known to cause adverse reactions. Accurate prediction of these side effects has the potential to expedite the drug discovery process, which is hindered because of high attrition of candidate drugs due to adverse drug reactions. The complex nature of mechanisms involving interaction of drugs with cellular processes makes it challenging to model this phenomenon. Availability of empirical data of drug features and known side effects provides a basis for building data-driven models aimed at prediction of side effects.

SIDER provides one of the best-annotated, manually curated data linking drugs to their adverse reactions. Over time, SIDER data has grown from an information repertoire of 888 drugs associated with 1450 side effects [1] to that of 1430 drugs associated with 5880 side effects [2]. Despite this growth in SIDER repertoire, these data come with some inherent problems. These side effects information, extracted from drug labels and post-marketing surveys, are reported with redundancy in their description. For example ‘Abdominal Pain’ and ‘Abdominal Discomfort’, ‘Choking’ and ‘Choking Sensation’ which could be considered equivalent are presented as separate entries. Prediction of side effects based on data similar to that from SIDER remains an intractable multi-label, multi-class problem. Further, the confusion matrix is unbalanced with far too many true negatives (missing drug-side effects associations) as compared to true positives (reported drug-side effects associations).

**Fig 1:**
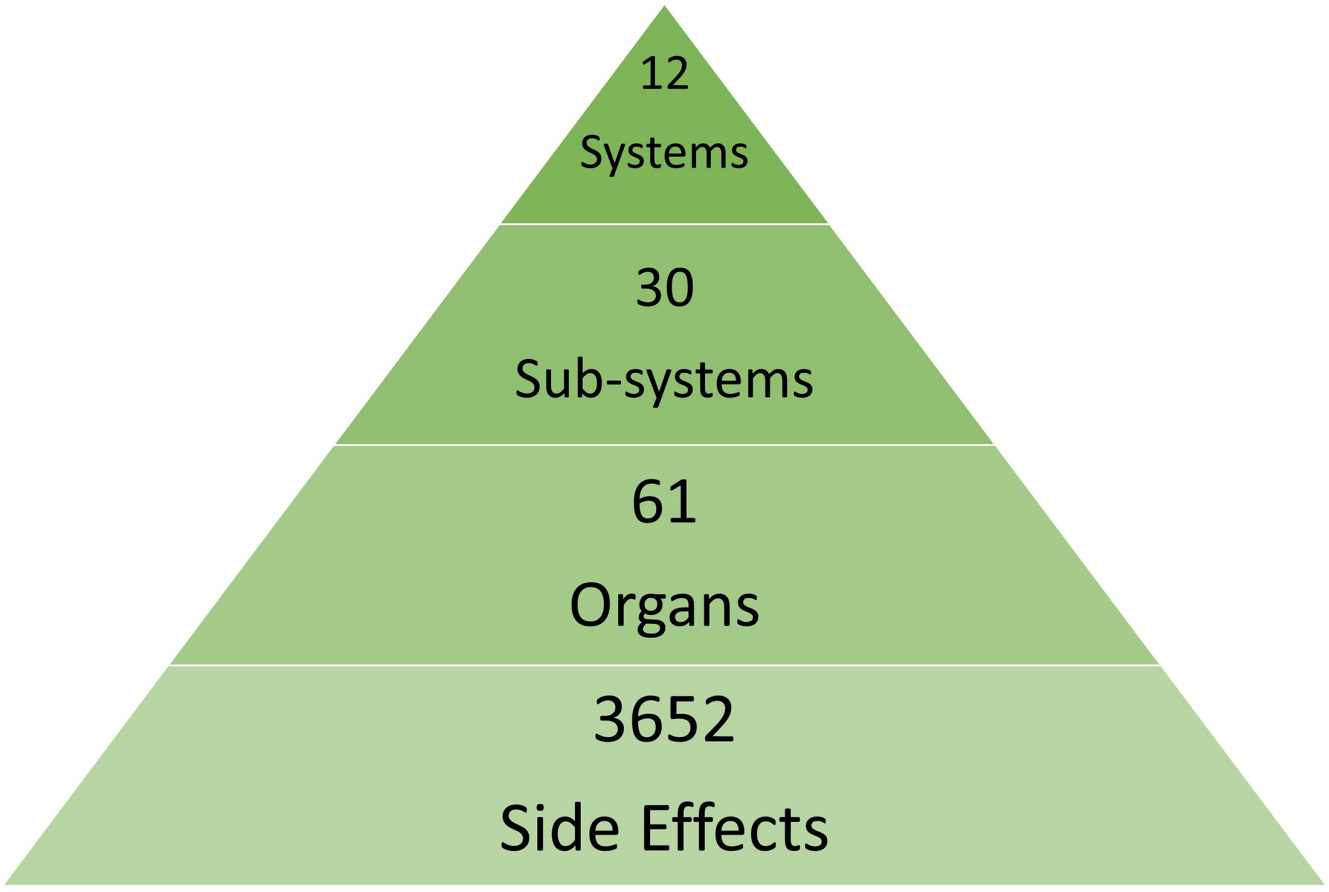
The hierarchical coarse-graining schema for anatomical aggregation of side effects to address redundancy and to achieve improved, meaningful classification.

**Fig 2:**
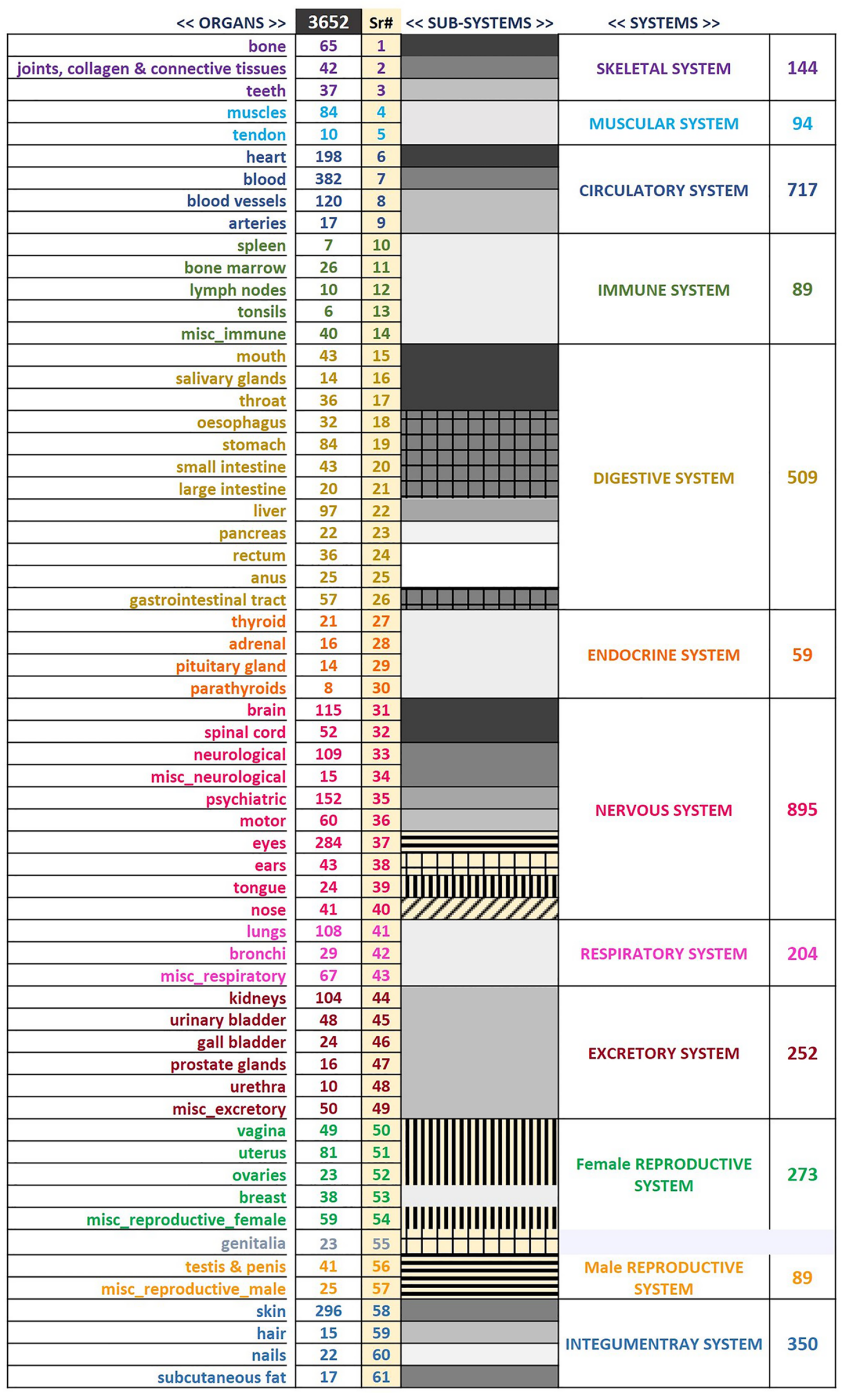
The schema for hierarchical anatomical classification of side effects by aggregating them into their associated organs, sub-systems and systems.

To address the problem of degeneracy in side effect labels, we implemented a strategy for hierarchical aggregation of side effects based on their anatomical presentation (Fig 1). Such a systematic aggregation of side effects was aimed at creating a coarse-graining of side effects into 61 organs, and further into 30 sub-systems and finally into 12 systems (Fig 2). We show that the classification performance can be improved with biologically meaningful coarse-graining. We suggest that our schema for aggregation of side effects could be effectively used for multi-level prediction of drug side effects.

Other than the problems of redundancy in side effects classes, appropriate evaluation of classification is a key problem. Early efforts towards implementation of classification algorithms on data from SIDER have exploited various drug features for predicting side effects [3–7]. These studies have relied on features such as target interactions and chemical similarity [8], network properties of the protein interactome of drug targets [7], information of targets and gene ontology [3, 6], and chemical structures and protein sequences apart from chemical similarities [4]. These encompass an array of features computed from drugs as well as those enumerated from the target space.

Regardless of the nature of information embedded in models and specifics of the classification strategy used, these studies evaluated the classification performance using AUC and/or accuracy. Given the skewed nature of data, relying on these metrics leads to misplaced confidence in the ability to correctly classify side effects with inflated values of AUC/accuracy. In the presence of data imbalance, it makes more sense to use metrics that account for ‘recall’ knowing that the data has a skewed representation of true negatives. We would like to highlight this ‘mismeasure of performance’ while working with side effects data and suggest the use of a balanced measure that carefully factors ‘recall’ apart from ‘precision’.

A few studies have highlighted this problem while using the SIDER data, and have used AUPR other than AUC [9–12]. Consistently across these studies, which have implemented k-nearest neighbor-based classifiers as well as methods based on canonical correlation analysis, while the models returned high AUC, they always performed poorly against AUPR. This further strengthens our argument for the use of a balanced measure that accounts for skewed nature of adverse drug reactions data. We argue that in the presence of over-representation of true negatives, a combination of AUC and F-score is a far more meaningful metric for evaluation of the classifier performance. In this study, we use a metric with equal contribution from AUC and *F*_2_ score (Weighted Performance).

Thus, to address the problem of redundant side effects labels we implemented a coarse-graining schema for their hierarchical aggregation based on anatomy. We also suggest the use of a balanced measure that factors in the AUC as well as F-score. Using 2D and 3D physicochemical properties of drugs as features, Random Forest was identified as the classifier with the best performance across all levels of coarse-graining. We also show that performance achieved by random aggregation at each level of coarse-graining is inferior to that obtained from aggregation rooted in anatomy. This suggests that our schema of anatomical segregation of side effects addresses data redundancy and accounts for the imbalance. In summary, our study presents a strategy for prediction of side effects by hierarchically aggregating them on the basis of anatomy.

## Methods

### Data collection from SIDER

Data of drug side effects were collected from SIDER 4.1 (http://sideeffects.embl.de, June 2017). These data comprised of 1430 drugs, 5868 side effects, and 139756 pairs of drug-side effects. Data from following three files obtained from SIDER were used for our analysis: meddra_all_se.tsv.gz, meddra_freq.tsv.gz, meddra_all_indications.tsv.gz. According to MedDRA (Medical Dictionary for Regulatory Activities), adverse reactions can be described at five different levels of concepts: System Organ Class (SOC), High Level Group Term (HLGT), High Level Term (HLT), Preferred Term (PT), and Lowest Level Term (LLT). SIDER provides the details of side effects at the levels of LLT and PT. While the specificity of an adverse reaction increases as one moves down the hierarchy, LLTs are extensions of PTs describing synonyms, lexical variants, quasi-synonyms or sub-elements of the same medical concept. Hence in our studies, information was considered at the level of PT by discarding LLTs (along with the null entries), as PTs are necessary and sufficient to characterize and differentiate single medical concept associated with a side effect [13]. With this filter, our data was reduced to 4314 side effects (linked to 1430 drugs) for which PT labels were available creating a new curated set of 136655 drug-side effect pairs. We used STITCH Identifiers (Search Tool for Interaction of Chemicals) of drugs for obtaining chemical properties of drugs. Starting with the flat STITCH ID, using Discovery Studio 4.1 we obtained an array of 330 2D and 57 3D properties, which were incorporated as drug features while building classifiers. The 2D properties quantify various molecular features related to atoms, bonds, rings, surface area, volume, molecular counts and electrostatic properties. The 3D properties represent aspects related to dipole, Jurs descriptors, energy, principal moments, shadow indices, surface area and volume. A full of list of these chemical features is given in S1 Table. The final curated data was then split into training and testing sets (80:20).

### Anatomical classification of side effects

Given that the data comprised of 4314 side effects presented in 1430 drugs, posing the side effects prediction problem in a standard manner runs into the stumbling block of performing multi-class multi-label classification with more classes than examples. To address this issue, we classified the side effects based on the organ or system in which they were presented (Figs 1 and 2). We manually classified each side effect into one of the 61 organs, into 30 sub-systems one level up and further into one of the 12 systems, thus creating a hierarchical coarse-grained classification rooted in anatomy. Among the organs with least number of associated side effects were tonsils (6), spleen (7) and parathyroids (8). Among the organs that were linked with largest number of side effects were blood (382), skin (296) and eyes (284). The statistics of side effects across different organs, sub-systems as well as systems is presented in Fig 2. Detailed structure of hierarchical anatomical coarse-graining schema is provided in S2 Table. A dynamic webpage implemented in D3.js illustrating the anatomical classification is available at http://cosylab.iiitd.edu.in/ADRhac.html.

Side effects that were too ambiguous to be linked to any particular organ were moved to the miscellaneous (MISC) category (e.g. application site discomfort, discomfort, disability, binge eating, breakthrough pain, chemical injury etc.). Also, the side effect was placed in the miscellaneous category belonging to an organ system, if it involved a number of organs from that system, affecting the system as a whole or part of the system that is not unambiguously identified as an organ (e.g. The side effect ‘hydroureter’ which refers to dilation of ureter has been placed in misc_excretory class). Among all the 12 systems, the following six classes have an additional miscellaneous category: Immune System, Nervous System, Respiratory System, Excretory System, Female Reproductive System, and Male Reproductive System. For the Digestive System side effects that did not affect any of the organs specifically but were generally affecting the gastrointestinal tract, were put under ‘gastrointestinal tract’. Thus, after ignoring the MISC class, we were left with a total of 3652 side effects that were categorized into 61 organs, which themselves belong to 12 systems. Such hierarchical classification of side effects based on their anatomical presentation facilitates prediction of side effects by effectively reducing dimensionality of the problem.

### Hierarchical schema for prediction of adverse reactions

After a meticulously implementation of anatomical classification of side effects at the level of organs, sub-systems and systems, we further predicted side effects on the basis of 2D and 3D drug features using various machine learning techniques. The adverse reactions were represented with a drug-side effects matrix of dimensions 1430 x 4314, in which drug indications were represented by binary one-hot vectors, with 1 depicting presence of adverse reaction and 0 representing a negative indication. Principal Component Analysis (PCA) was used to extract the first one hundred principal components (embodying 2D and 3D properties) that capture 98.4% of the total variance in data. These 100 components associated with each of the drugs, were further used for predicting the side effects.

We used following supervised classifiers for prediction of side effects at different levels of resolution (side effects, organs, sub-systems and systems): Logistic Regression (L2 Regularized), SVM (with radial basis function), kNN, GaussianNB and Random Forest. We implemented 5-fold cross-validation during training and used a range of metrics to assess the prediction performance on the test set: F2 score, AUPR, AUC, Precision and Recall. Further to improvise the performance, we optimized the classification task to build a soft-voting based ensemble model by using the three best-performing classifiers. Analysis of a binary classifier yields true positives (TP), true negatives (TN), false positives (FP), and false negatives (FN) as predicted over the test set and observable from confusion matrix. We implemented various measures of performance in this study: *Precision*, *Recall*, *F*2 *Score*, *AUC*.

$$\begin{array}{c} Precision=\frac{TP}{TP+FP} \end{array}$$

$$\begin{array}{c} Recall=\frac{TP}{TP+FN} \end{array}$$

$$\begin{array}{c} F2=\frac{5\cdot Precision\cdot Recall}{4\cdot Precision+Recall} \end{array}$$

Area Under the Curve from Receiver Operating Charaterstic analysis accounts for all possible thresholds that are used to measure a classifier’s performance and is defined as,

$$\begin{array}{c} AUC=\frac{True\;Positive\;Rate}{False\;Positive\;Rate} \end{array}$$

Due to the nature of the SIDER data with high class imbalance (See Section Assessment of classifier performance), we evaluated the performance of classifiers using a weighted measure that was calculated as the average of *F*2 *Score* and *AUC*.

Random control experiments were conducted to account for the improvement in performance due to random aggregation (coarse-graining) of side effects with no biological basis. In these experiments pseudo-organs, pseudo-sub-systems as well as pseudo-systems, matching in number and size of that of the anatomical organs, sub-systems and systems, respectively, were created. Statistics of performance measured with such pseudo-organs and pseudo-systems were computed based on 100 random experiments each.

All experiments reported in this study were performed in Python, a free high level object-oriented programming language that facilitates data analysis and machine learning through a powerful suite of analytical tools. Scripts and data are available on https://github.com/cosylabiiit/drugADR. Computations were performed on IIIT-Delhi servers.

## Results

### Unbalanced nature of SIDER data

**Fig 3:**
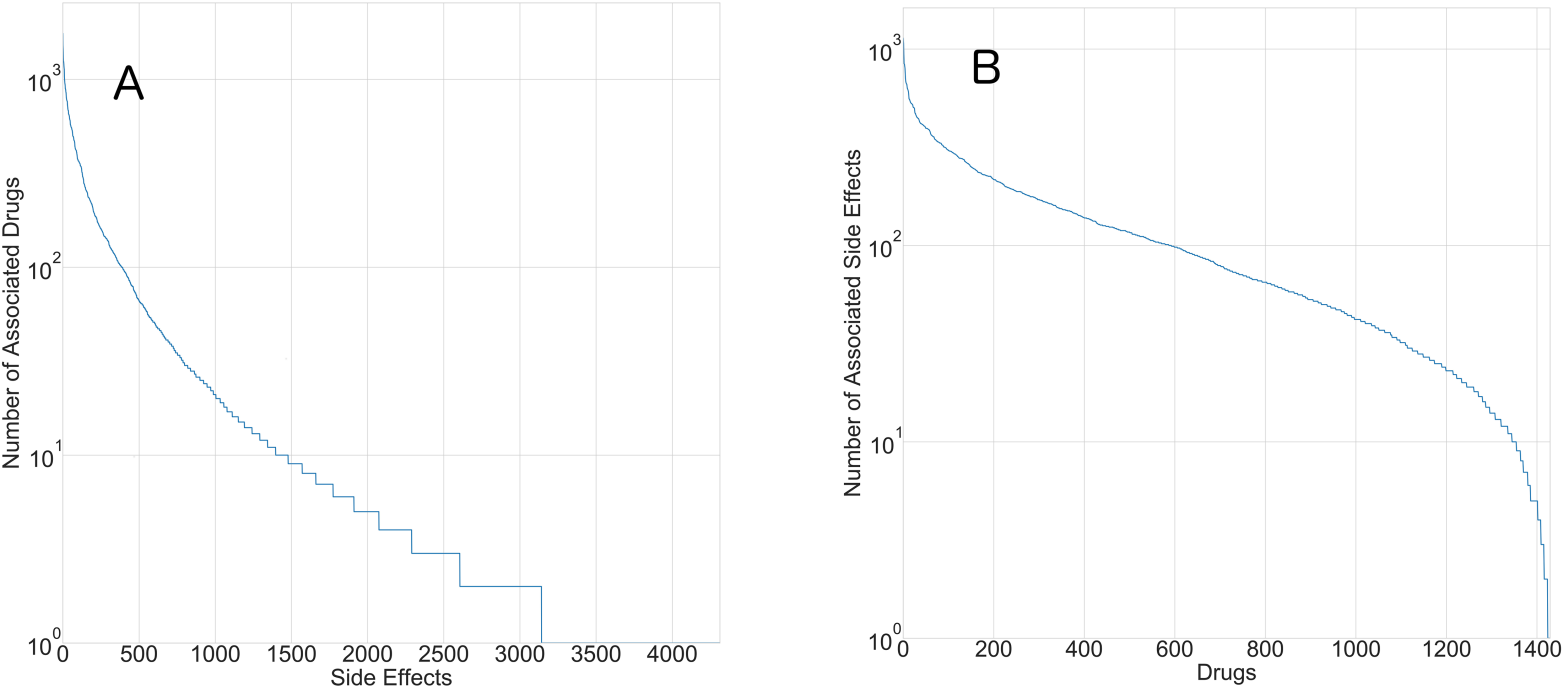
(A) Distribution of side-effects across drugs showing presence of drugs associated with exceptionally large number of side effects. (B) Distribution of drugs across side effetcs depicting presence of adverse reactions that are linked to large number of drugs.

Quality of results obtained via machine learning are contingent upon the nature of data, selection of relevant features as well as evaluation metrics. SIDER provides one of the most well-curated data of adverse drug reactions, facilitating their data-driven predictions [1, 2]. These data of drug side effects, meticulously curated from their drug labels, comprise of 1430 drugs and 5868 side effects. Associations between drugs and side effects were inhomogeneous and were loaded with drugs with exceptionally large side effects as well as frequently reported side effects. When seen from the perspective of drugs, the data were skewed with drugs that were presented with exceptionally large number of adverse reactions (Fig 3A). They also showed presence of side effects that were caused by exceptionally large number of drugs (Fig 3B). Also, these data were sparse with number of drug-side effects pairs far less compared to maximally possible. In the presence of such unbalanced data with large fraction (98%) of true negatives, any implementation of classification strategy needs to give more credence to the ability to ‘recall’. Therefore assessing classification performance using ‘accuracy’ as a measure could be misleading for such data with far too many labels.

While some studies have pointed out this mis-measure of performance [9–12], efforts towards building side effects classifiers have heavily relied on AUC-ROC as a measure of performance hitherto [3–8, 14–19]. Previous studies that enumerated AUC as well as precision-recall have reported a contrast in performance with high AUC and low precision-recall [9, 10, 12, 20]. With AUC nearly double that of AUPR/F-Score, these data clearly indicate the sensitivity of ROC analysis towards class imbalance [21].

### Assessment of classifier performance

With this premise, we intended to design a strategy that could be meaningfully implemented for the prediction of adverse reactions. Since, AUC-ROC when used alone is sensitive to a class imbalance [21], using it as the sole measure of a classifier’s performance does not suffice (Table 1). Hence, we chose to use an evaluation metric that assesses performance of a classifier on two fronts—a high AUC-ROC, which is a function of a threshold as well as a de-facto standard for measuring classifier performance, and a high F-score, which is an absolute point wise metric independent of threshold. Accordingly, we suggest the use of a balanced metric (such as Weighted Performance) to evaluate the classifier.

**Table 1:** Evaluation of classifier performance in response to AUC-ROC and F score (or any other point metric) in the presence of class imbalance. A metric requiring high F score as well as AUC-ROC provides a better measure of classification performance.

F Score	AUC-ROC	Classification Performance
Low	Low	Bad performance; altering threshold may help
Low	High	Bad performance; for extreme thresholds the performance may be satisfactory.
High	Low	Satisfactory performance for a specific threshold; however yields bad performance for other thresholds.
High	High	Good performance independent of threshold; A desirable criterion.

### Physicochemical properties as drug features

Various aspects of drug, and cellular entities that it interacts with, play a role in exhibition of adverse drug reactions. Earlier studies have considered various drug features (2D & 3D properties, drug substructures) as well as features such as drug targets, molecular pathways and GO terms for prediction of side effects [5, 8, 16, 19]. Among other features, 2D & 3D properties of drug are known to yield good prediction performance of side effects [22].

We compiled an array of physicochemical properties of drugs covering 2D (315) and 3D (57) features for our studies. These chemical attributes include a wide range of properties of potential relevance in specification of side effects. The features include molecular weight, hydrogen bond acceptors/donors, number of double bonds, energy, number of aromatic rings, molecular solubility, logP etc. As a standard procedure used for reduction of features [23, 24], we employed Principal Component Analysis to identify key features that primarily contribute to the variability in data. We observed that the first 100 components account for 98.4% of the total variance in data, and hence these were used as drug features for the remainder of our studies. For PCA results please see S1 Fig.

### Hierarchical anatomical coarse-graining of side effects

We propose a strategy for hierarchical coarse-graining of side effects rooted in their anatomical presentation for effective classification of side effects (Fig 1). Starting from the list of all side effects provided in SIDER, we first bundled them into 61 organs to which these adverse reactions are known to be associated with (Fig 2). Further the side effects were coarse-grained to bundle them in to 30 sub-systems and 12 anatomical systems. We hypothesize that aggregating side effects based on organ systems creates meaningful classes compared to random aggregation.

**Fig 4:**
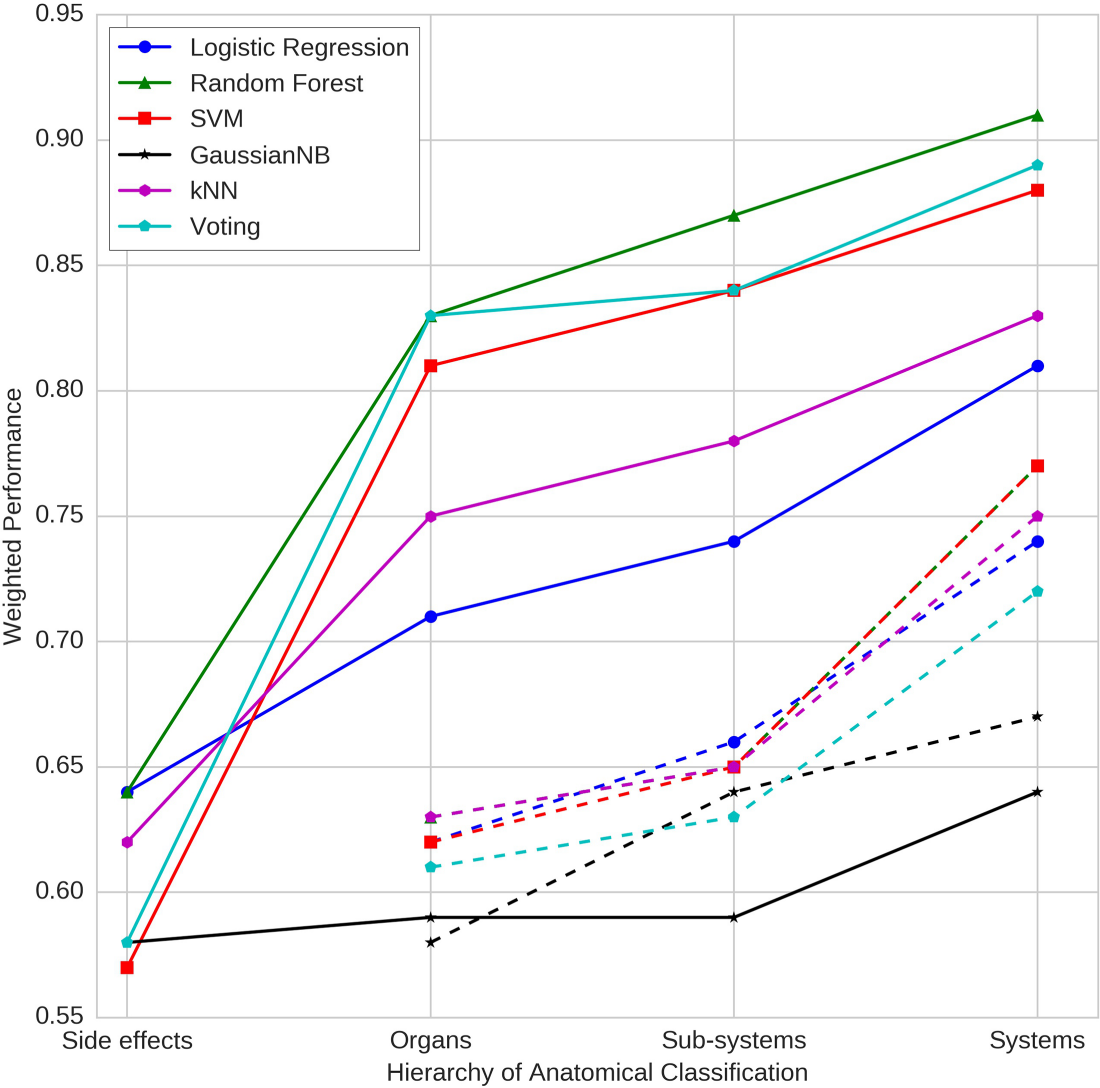
Comparison of prediction performance of classifiers at different levels of coarse-graining. Aggregation of side effects rooted in anatomy significantly improves classification performance compared to arbitrary aggregation. Random Forest model yielded best performance across the anatomical hierarchy.

Fig 4 depicts the performance of different classifiers at different levels of coarse graining of side effects: ‘Side Effect Level’ refers to no coarse graining (by considering each side effect as an independent label/class); ‘Organ Level’ refers to the organ classes where side effects have been aggregated based on their anatomical presentation; ‘Sub-system Level’ refers to a higher level coarse-graining into sub-systems; ‘System Level’ refers to further aggregation of side effects based on the anatomical system to which organs belong. Performance of each classifier was evaluated on the basis of ‘F2 score’ that is weighted towards ‘Recall’ as well as the AUC. This ‘Weighted Performance’ is a balanced metric which attempts to overcome the problem of the incorrect assessment of performance in the presence of data imbalance (Table 1).

**Fig 5:**
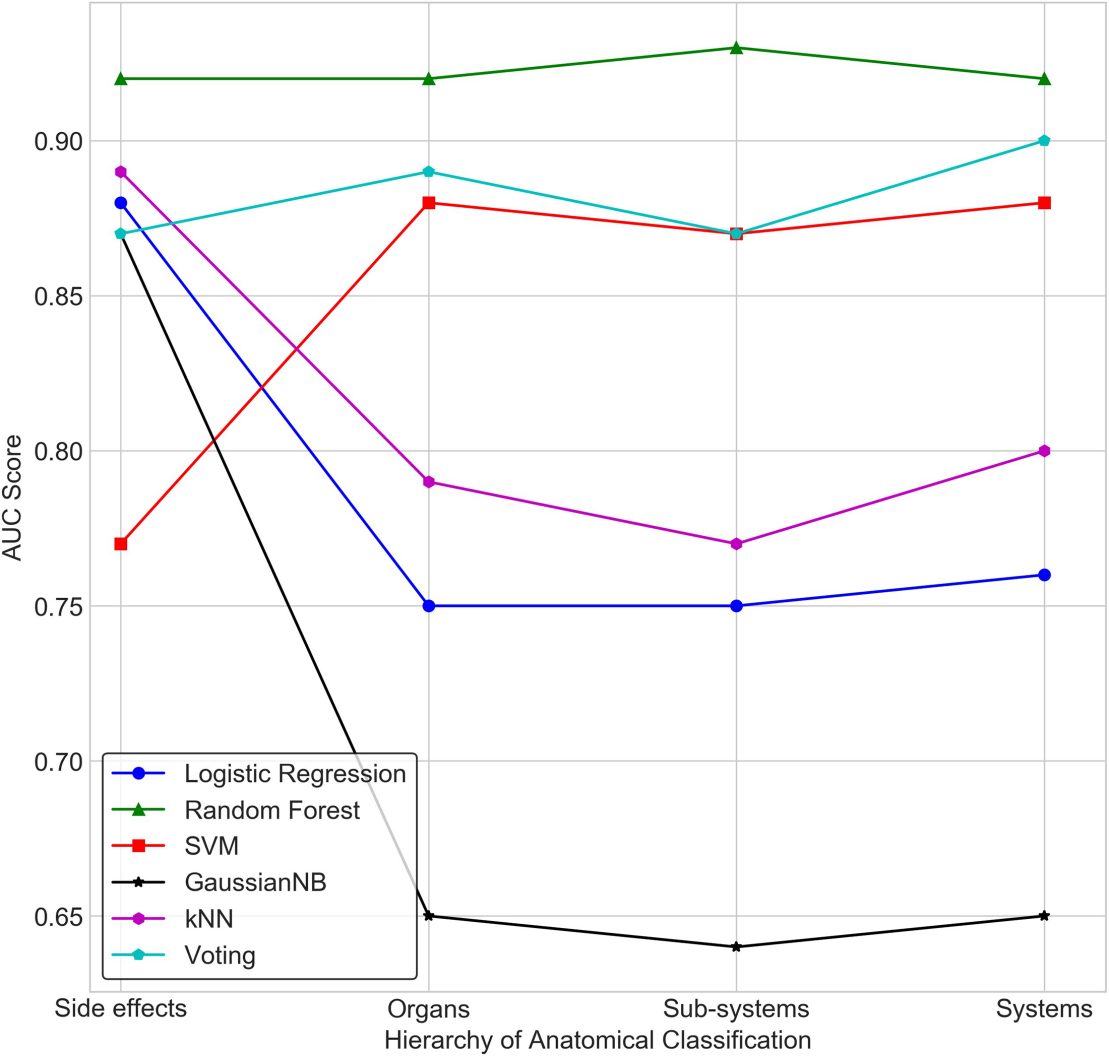
Comparison of prediction performance of classifiers in terms of AUC score, at different levels hierarchy.

**Fig 6:**
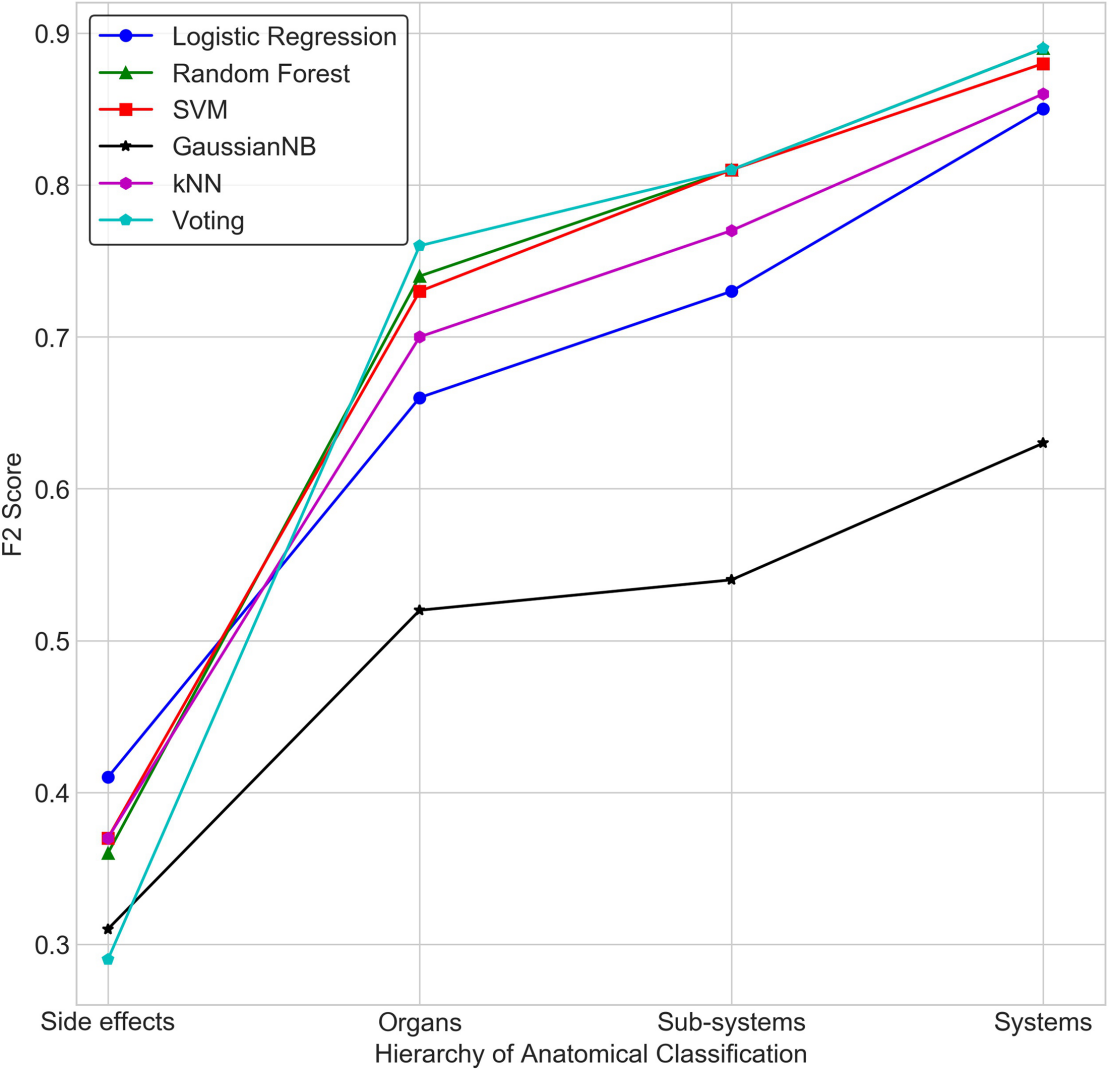
Comparison of prediction performance of classifiers in terms of F2 score, at different levels hierarchy.

As argued earlier, at the level of side effects where each of the 3652 side effects is a label/class, every classifier shows very good performance in terms of AUC (Fig 5), but fails with the recall measure (Fig 6). This is reflected in the Weighted Performance, which is in the range of 0.57 to 0.64 across various classifiers.

With aggregation of side effects on the basis of organs, the performance improves consistently across classifiers as well as each of the metric used (range: 0.58–0.81). As anticipated, unable to capture non-linearities in the data, Gaussian Naïve Bayes performs poorly. Bundling side effects at the level of sub-systems further enhances the performance suggesting the utility of coarse-graining of side effects based on anatomy. Higher level aggregation to reduce the number of classes to 12 broad systems, enhances the performance further. To ensure that the training is free from any bias, we employed the leave-one-out cross validation strategy on the two best performing models- Random Forest Classifier and the Voting Ensemble. The comparison of results are available in S3 Table.

**Fig 7:**
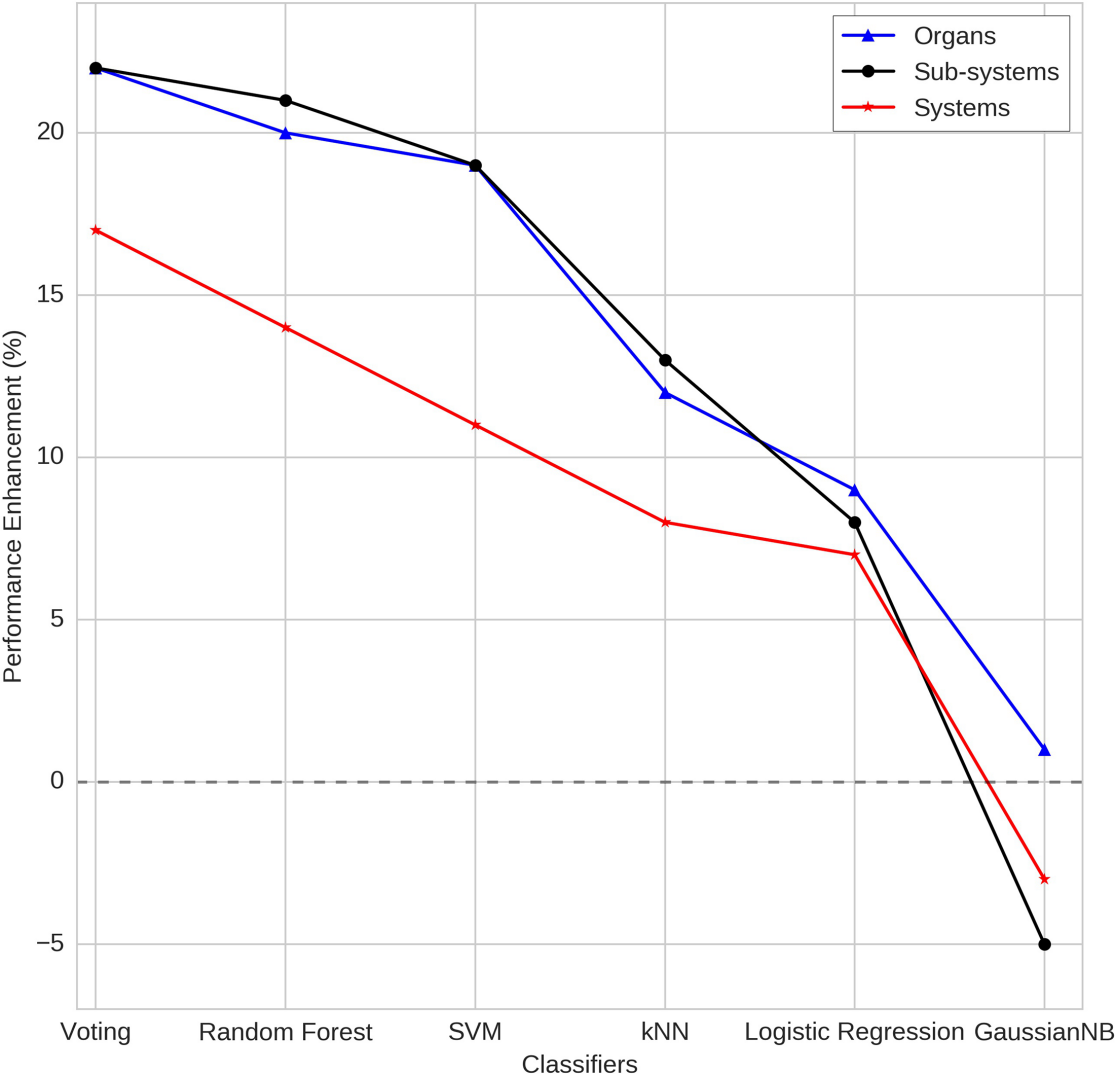
Enumeration of performance enhancement at different levels of coarse-graining as compared to random aggregation.

Furthermore, at every level, the random control refers to experiment in which the side effects were aggregated randomly (as opposed to that on the basis of organ, sub-systems and anatomical systems). While the act of aggregation itself expectedly enhanced the performance, the improvement was only marginal as compared to meaningful aggregation rooted in anatomy. Fig 7 clearly depicts the relevance of anatomical coarse-graining strategy. The model implementing soft-voting based ensemble emerged with best enhancement in classification performance across the level of coarse-graining, followed by Random Forest, SVM, kNN and Logistic Regression. Gaussian Naïve Bayes yielded performance that was worse than random aggregation. In summary, these results support our hypothesis and establish the utility of hierarchical anatomical coarse-graining strategy towards achieving improved performance of side effects classification.

## Summary and conclusions

Prediction of adverse drug reactions is a key problem in drug discovery process. Data-driven studies founded in empirical data have the potential to address this challenge. SIDER is one of the most heavily used dataset towards this objective, and has been widely implemented for identification of drug features as well as building machine learning models for the classification of side effects. We highlight the inherent imbalance in these data that render studies solely relying on performance metrics such as AUC irrelevant. We argue for the use of metrics that are robust to class imbalance and present a hierarchical anatomical classification schema which aggregates side effects into organs, sub-systems and systems. With the help of a weighted performance measure, using 5-fold cross-validation we show that this schema facilitates effective prediction of side effects. Such a manually curated partitioning of side effects into different anatomical classes can also serve as a basis of future studies towards prediction of adverse reactions and identification of key features linked to specific organ systems.

The objective of this study was to find ways of addressing multi-label, multi-class classification problem. While in this study we have used 2D & 3D physicochemical properties, one may implement this strategy with the help of other features as well. Another potential future direction is seeking for automated strategies for aggregation of side effects. Beyond manual curation of side effects, it would be interesting to see whether ontology-based strategies could be effective for coarse-graining. Our study provides a unique view of side effects rooted in anatomy and can pave the way for calibrated expert systems for multi-level prediction of side effects.

## Supporting information

S1 Fig Results of Principal Component Analysis on 2D and 3D drug properties. (TIF)

S1 Table Full list of 2D and 3D chemical properties used as drug features. (XLSX)

S2 Table Detailed structure of hierarchical anatomical coarse-graining schema. (XLSX)

S3 Table Comparison of cross validation & test set results on best performing models. (XLSX)
