# Supplementary figures and images for: A hierarchical anatomical classification schema for prediction of phenotypic side effects
Source: PLoS One. 2018 Mar 1;13(3):e0193959. doi: 10.1371/journal.pone.0193959 (PMC5832387; doi:10.1371/journal.pone.0193959)

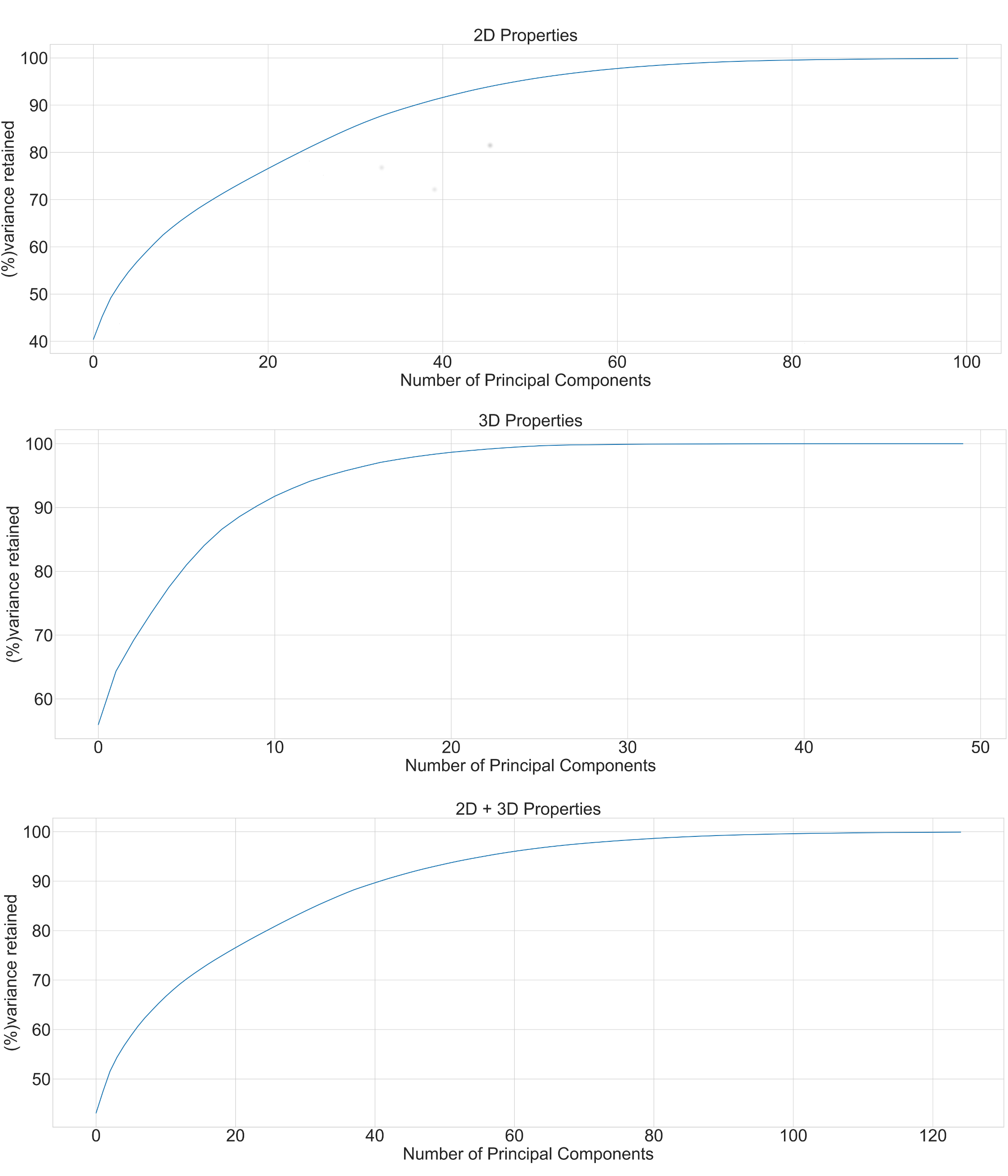

Supplement: S1 Fig — (TIF) [file pone.0193959.s001.tif]
